# The socio-ecological resilience and sustainability implications of seafood supply chain disruption

**DOI:** 10.1007/s11160-023-09788-1

**Published:** 2023-06-13

**Authors:** Roshni C. Subramaniam, Mélodie Ruwet, Fabio Boschetti, Simon Fielke, Aysha Fleming, Rosa Mar Dominguez-Martinez, Éva Plagányi, Peggy Schrobback, Jessica Melbourne-Thomas

**Affiliations:** 1 CSIRO Environment, Hobart, TAS 7000 Australia; 2 School of Government and International Relations, Griffith University, Queensland, 4222 Australia; 3 CSIRO Environment, Crawley, WA 6009 Australia; 4 CSIRO Environment, Dutton Park, QLD 4102 Australia; 5 School of Earth and Environmental Sciences, University of Queensland, Queensland, 4067 Australia; 6 CSIRO Environment, St Lucia, QLD 4067 Australia; 7 CSIRO Agriculture and Food, St Lucia, QLD 4067 Australia; 8 Centre for Marine Socioecology, University of Tasmania, Hobart, 7000 Australia

**Keywords:** Seafood supply network, Socio-ecological resilience, Sustainability, Shocks, Complex adaptive systems, Equity

## Abstract

Remaining resilient under disruption, while also being sustainable, is essential for continued and equitable seafood supply in a changing world. However, despite the wide application of resilience thinking to sustainability research and the multiple dimensions of social-ecological sustainability, it can be difficult to ascertain how to make a supply chain both resilient and sustainable. In this review, we draw upon the socio-ecological resilience and sustainability literature to identify links and highlight concepts for managing and monitoring adaptive and equitable seafood supply chains. We then review documented responses of seafood supply networks to disruption and detail a case study to describe the attributes of a resilient seafood supply system. Finally, we outline the implications of these responses for social (including wellbeing and equity), economic and environmental sustainability. Disruptions to supply chains were categorised based on their frequency of occurrence (episodic, chronic, cumulative) and underlying themes were derived from supply chain responses for each type of disruption. We found that seafood supply chains were resilient when they were diverse (in either products, markets, consumers or processing), connected, supported by governments at all scales, and where supply chain actors were able to learn and collaborate through trust-based relationships. With planning, infrastructure and systematic mapping, these attributes also can help to build socio-ecological sustainability and move towards more adaptive and equitable seafood supply.

## Introduction

### Background

**Fig. 1:**
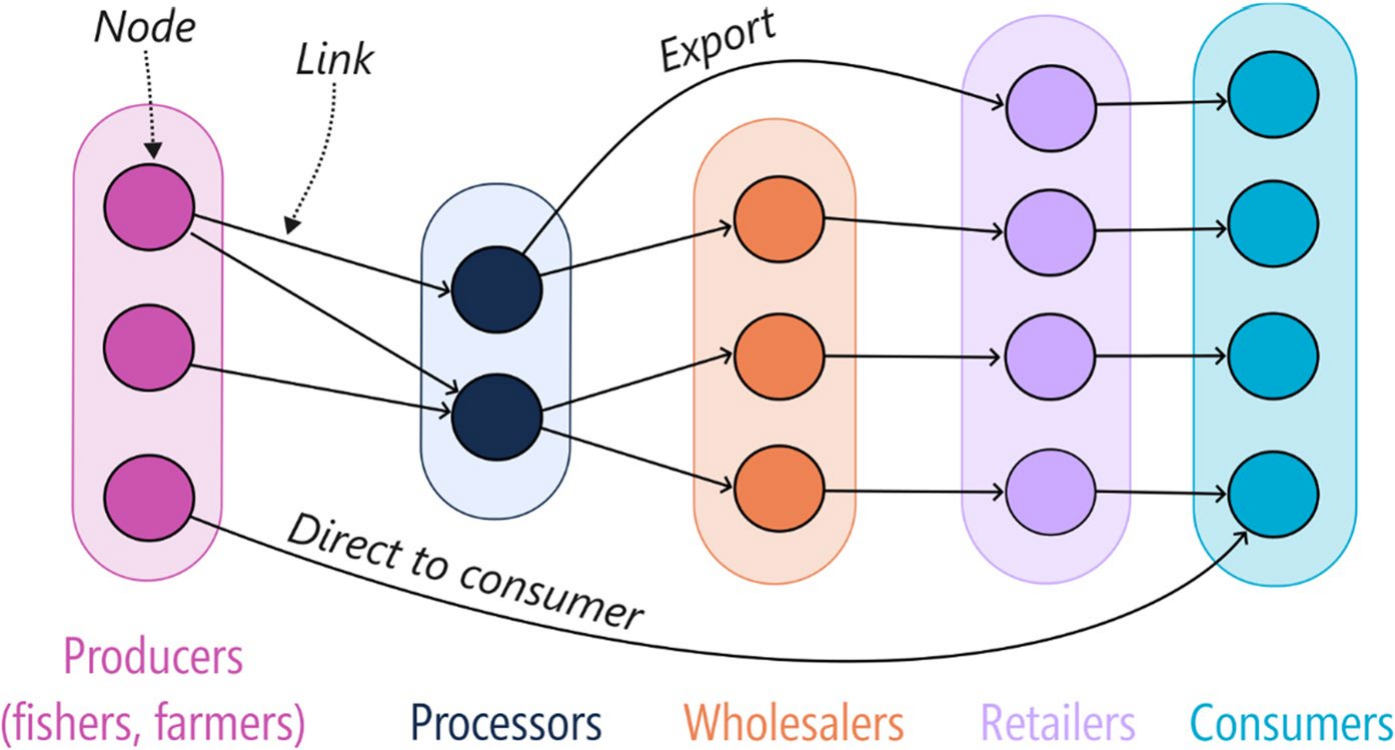
Generic seafood supply chain network (SSCN) for a harvested species where each node represents an element, and links (arrows) represent the direction of seafood supply between nodes. Example links described in the text are labelled

Seafood supply chain networks (SSCNs) are complex socio-ecological systems, connecting marine ecosystems to countries, regions, businesses and markets. In comparison to value chains, supply chains relate to the supply of the product to the consumer rather than the value adding processes created by a business (Lim-Camacho et al. 2021). Seafood supply is harvested from the ecosystem or tank and pond-based aquaculture systems by producers (i.e., fishers and farmers) and flows to the consumer via multiple intermediaries such as processors and wholesalers (Pullman and Wu 2021). Transport logistics and infrastructure are key elements that support the connections between different nodes (links in Fig. 1). Each stage of the supply chain may have multiple nodes that can represent multiple farms, fishing locations or operators at the supply end, or multiple retailers at the consumer end (Schrobback and Rolfe 2021) (Fig. 1). For example, some supply chains export more of a fished species than they sell domestically, bypassing parts of the SSCN (Fig. 1). Roles of supply chain actors can also overlap, for instance, where producers also supply directly to the consumer (Fig. 1).

Seafood is an important and highly traded food source with ~ 34% of the global fisheries and aquaculture production volume exported in 2020 (FAO 2022). Fisheries and aquaculture are key to the livelihoods of many communities and nations (FAO 2022). Moreover, seafood is a vital source of micronutrients and essential fatty acids in coastal Indigenous communities (Cisneros-Montemayor et al. 2016) and small-scale fisheries and low and middle-income nations, which are highly connected to international trade (Crona et al. 2016; Nash et al. 2022a). Seafood production is meeting growing global demand for food and protein (Farmery et al. 2022). However, climate change and other anthropogenic pressures (e.g., geopolitics, market changes) create disruptive events that hamper their potential to meet projected demands for healthy and affordable diets (FAO 2022).

Seafood supply chains can be extensively connected to worldwide markets, hence their vulnerability to disruptions occurring at multiple spatial and temporal scales not only increases through exposure, but can also have cascading and disproportionate impacts across the supply chain to seafood dependent communities (Bassett et al. 2021 and references therein; de la Puente et al. 2022). Shocks to food supply systems are increasing in frequency and severity (Gephart et al. 2017; Cottrell et al. 2019) and addressing the vulnerabilities within supply chains is key to securing the global sustainability of seafood (Lim-Camacho et al. 2014). Studies on seafood system resilience have modelled SSCN using network-based approaches (Plagányi et al. 2014, 2021) or documented responses to disrupted seafood supply (e.g., Ogier et al. 2021; Love et al. 2021). The spread of COVID-19 has notably exposed many vulnerabilities in seafood supply chains (e.g. loss of markets and transport) (Bassett et al. 2021), which are informing new research on SSCN resilience and sustainability (e.g. Plagányi et al. 2021). However, as SSCNs operate within different contexts and are connected across scales, specific methods or adaptation options reported may not be transferable. Therefore, a generalised and holistic approach is needed to build resilience.

Prior studies of sustainable seafood supply have mostly focused on the production stage of the supply chain (Simmance et al. 2022) or outcomes for environmental sustainability (Denham et al. 2015; Simmance et al. 2022). Recent work has shed more light on sustainability in seafood systems by addressing needs for equity, socio-economic sustainability, wellbeing and meeting the SGDs (Farmery et al. 2022). However, it still unclear how sustainability can be achieved under disruption while also meeting current and future demands (Simmance et al. 2022). Thus, pathways to equitable and adaptive seafood supply are interlinked with building socio-ecological resilience and sustainability, with a key challenge of addressing the local and global scales at which these processes occur (Cockburn et al. 2020). This is done by first defining resilience and sustainability within the context of seafood supply chain disruption, and then considering the attributes of each concept that comprise an equitable and adaptable seafood supply system.

### Aims

**Fig. 2:**
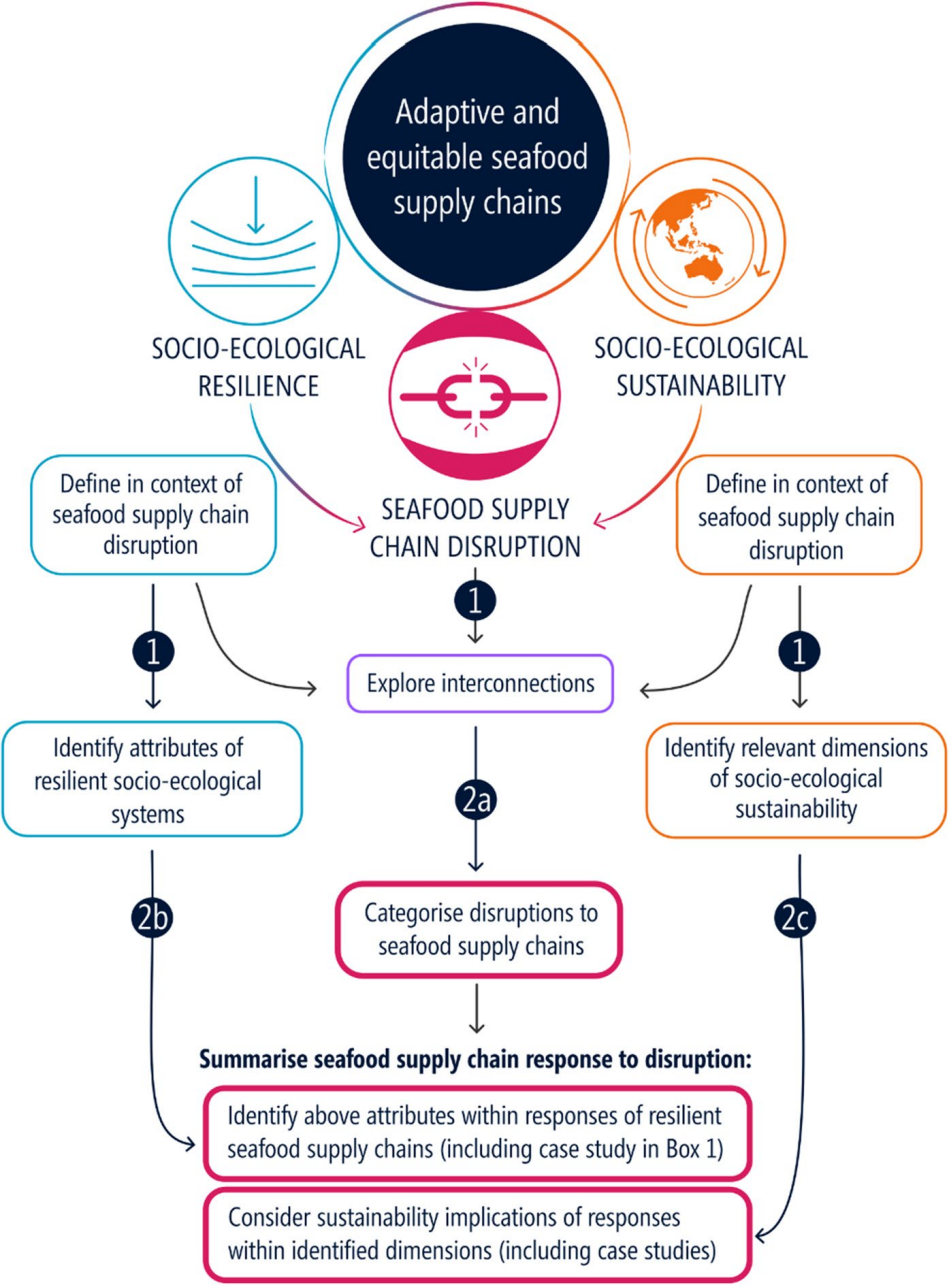
Schematic to show layout of this review where numbers correspond to the aims of the study. Arrows indicate direct links of conceptual elements to the new contributions of this paper to the literature. Thick red boxes indicate our contributions to the literature

Our aims were to (i) identify the links between socio-ecological resilience and sustainability that are crucial for managing and monitoring adaptive and equitable SSCNs (Fig. 2); and (ii) assess the relevance of these concepts for building adaptive and equitable seafood supply chains (Fig. 2). Specifically, we:

- Categorised disruptions to SSCNs;
- Reviewed SSCN responses to disruption and provide a case study to identify resilience-building strategies for SSCNs within different contexts and;
- Considered the socio-ecological sustainability implications of these responses to suggest a path forward for ensuring that adaptive responses are also equitable and sustainable.

We address these aims through a mixed methods review (Grant and Booth 2009), where the first component is a brief narrative synthesis of the resilience and sustainability literature within the context of seafood supply chain disruption (Aim 1, Fig. 2) and the second is a qualitative synthesis of seafood supply chain responses to disruption (Aim 2, Fig. 2). Our findings contribute to an improved understanding of how the complementary concepts of sustainability and resilience apply in the context of seafood supply chain disruption. This is fundamental for the management of seafood supply chains by its stakeholders (e.g., producer associations, government, retailers).

## Methods

Our mixed methods review (Grant and Booth 2009) was semi-structured, where we used search terms on Google Scholar to find peer-reviewed papers and reports, then followed references within these initial papers to find relevant concepts and information. We used the following search terms for the first component: “socio-ecological resilience”, “supply chain sustainability”, “supply chain resilience”, “supply chain disruption”, “seafood supply chain”, “food system”, “seafood system resilience”, “seafood system sustainability”, “seafood supply network”, “fisheries resilience” and “sustainable fisheries”. From the papers discussing socio-ecological resilience and sustainability, we identified the concepts and attributes that were relevant to seafood supply chain disruption (Aim 1, Fig. 2). The following additional search terms were used for the second component of this review: “COVID-19 impacts + seafood”, “seafood disruption”, “seafood production” + “shock”, “seafood supply chain disruption”. We used papers discussing responses to disrupted seafood supply and/or seafood system resilience to categorise disruptions based on the frequency and impact of the disruption. Next, we identified the attributes of the SSCN that enabled resilience (Aim 2, Fig. 2). Finally, we outline how resilience attributes can also build sustainability and refer to the literature for examples.

## Results and discussion

### Links between resilience and sustainability for supply chain disruption

Sustainability is understood as meeting the demands of the current generation without compromising resources for future generations (intergenerational equity) (World Commission on Environment and Development 1987). Seafood supply and the interactions within and along the supply chain support up to half of the seventeen Sustainable Development Goals (SDG) (United Nations 2015; Blanchard et al. 2017). Seafood supply chains support goals to improve livelihoods (SDG 1), health and wellbeing (SGD 3), equality (SDG 10) and food security (SDG 2, 12), all of which are enabled by life below water (SGD 14) and climate action (SDG 13) (Blanchard et al. 2017). Here we view sustainability through a socio-ecological lens, where sustainability is informed by the SDGs, but also considers the ecological, economic and social dimensions within the seafood context (marine environments, seafood-based industries and seafood dependent communities, respectively).

Closely linked to sustainability is resilience, which describes the ability of the system to respond to external impacts. Linking resilience to sustainability is recognised as important for managing socio-ecological systems in an uncertain and changing world (Reyers et al. 2022); building resilience alone could for example, result in a system that is effective at responding to disruption but does not achieve sustainability goals (Xu et al. 2015). In the seafood context, resilience and sustainability together implies, that long-term human activities in the socio-economic dimension (e.g., fishing) does not impact the marine ecosystem even if the supply chain activity exceeds a threshold (or vice versa) (Xu et al. 2015).

Interactions along SSCNs may be linear or nonlinear, and one directional, or have thresholds and delayed feedbacks. For example, delays to shipments of live or frozen seafood can lead to waste due to limited storage, or reduced quality and customer dissatisfaction (Graziano et al. 2018; Bennett et al. 2020). Additionally, external events (e.g., stock dynamics) can influence supply chain operations yet are not usually holistically connected to them (Simmance et al. 2022). Knowledge, ownership and regulation are compartmentalised while the disruptions that SSCNs face are interdependent (Cockburn et al. 2020; Novak et al. 2021). Engineering and ecological resilience concepts have been used to characterise supply chains by assuming equilibrium states; however, they tend to exclude the features necessary for capturing the dynamic and adaptive nature of seafood supply chains. These dynamic features are more embedded in complex adaptive systems research (Novak et al. 2021; Reyers et al. 2022).

The concept of socio-ecological resilience is better suited for implementing resilience and sustainability into complex adaptive systems (Novak et al. 2021; Reyers et al. 2022). Socio-ecological resilience for supply chains is defined as the ability of the system to adapt in response to multiscale disruption and maintain function (Carpenter et al. 2001; Novak et al. 2021). Resilience thinking can be targeted towards identified shocks, where part of the system is resilient to a particular disruption, or applied generally by identifying the characteristics of a system that determine its ability to cope with unidentified shocks (e.g., Walker et al. 2009). Due to the uncertain and complex nature of disruptions that can impact all stages of a SSCN, and the complexity of interactions within SSCNs, we suggest that building general resilience is better suited for SSCN management and sustainability.

Biggs et al. (2012) propose seven principles for enhancing socio-ecological resilience: diversity and redundancy, slow variables and feedbacks, connectivity, an understanding of complex adaptive systems, learning and experimentation, broad participation and polycentric governance (Table 1). These principles provide a holistic understanding of the system and outline options for building resilience. Similar attributes have been defined within ecological, socio-economic and governance domains to confer climate resilience for holistic fisheries management (Mason et al. 2022) and to describe properties of resilient supply chain firms (Wieland et al. 2023; Roque Júnior et al. 2023) (Table 1). The seven principles support the shifts needed to better integrate resilience into sustainable development for complex adaptive systems as they focus on understanding context, nonlinearity, and the dynamic relationships, scales and capacities existing within complex adaptive systems and by extension, SSCNs (Reyers et al. 2022; Wieland et al. 2023).

**Table 1:** System attributes for building socio-ecological resilience and SSCN equivalents

Attributes (Biggs et al. 2012)	Definition	SSCN equivalents
Diversity and redundancy	Maintaining diversity and redundancy: • Diversity refers to the variety, balance and disparity of elements and responses to disruption (response diversity) • Redundancy describes elements of the system that compensate for the loss of other elements	• Agility–the ability to respond quickly, smoothly and cost-effectively to shocks in supply or demand (Shekarian and Mellat Parast 2021; Roque Júnior et al. 2023). For example, diversifying production • Flexibility–flexible operations (Shekarian and Mellat Parast 2021; Roque Júnior et al. 2023), harvesting strategies or ecological diversity (Mason et al. 2022)
Connectivity	Managing the connections between elements in the system (nodes in Fig. 1). For example, increased connectivity between social groups increases information sharing and trust. Conversely, overly connected systems can decrease resilience	• Visibility–ability to map supply chains and pathways to consumers (Roque Júnior et al. 2023) • Organisation–interactions between social and ecological dimensions (Mason et al. 2022)
Slow variables and feedbacks	Managing slow variables and feedbacks: • Slow variables refer to regulating variables such as climate or legal systems • Feedbacks are changes in a variable or interaction that strengthens or reduces subsequent changes of the same type (e.g., lack of information sharing due to competition, Fleming et al. 2014)	Seafood supply chains that move live or fresh product are more vulnerable to short-term disruptions Additionally, the timing of a disruptive event is important. For example, supply chains may be less impacted by a disruption that occurs outside of the fishing season than during (Ogier et al. 2021)
An understanding of complex adaptive systems	Acknowledges that: • Systems are continually evolving, adapting and are linked across multiple spatial and temporal scales • Uncertainties exist • Holistic approaches are needed	• Knowledge of supply chain structure and cross-scale interactions (Novak et al. 2021) • Scales–climate-resilient management approaches that consider temporal and spatial scales (short-term, mid-term, long-term) (Holsman et al. 2017)
Learning and experimentation	Enables socio-ecological systems to adapt and remain resilient under uncertainty, change and surprise (e.g., monitoring and knowledge sharing)	• Collaboration (Shekarian and Mellat Parast 2021; Roque Júnior et al. 2023) • Learning (Mason et al. 2022)
Broad participation	Active engagement of relevant stakeholders in management and governance	• Collaboration (Fleming et al. 2014; Shekarian and Mellat Parast 2021; Roque Júnior et al. 2023) • Agency–the capacity to negotiate and make decisions (Mason et al. 2022)
Polycentric governance	Governing authorities at different scales (local, regional, national, international) working together when relevant	Ways in which fisheries management can enable resilience (Mason et al. 2022)

Ultimately, seafood supply is managed by people. People need to have the capacity to make decisions that lead to resilient and sustainable seafood supply systems. This not only relates to the resources available to communities and individuals to support adaptation (through equity) but also an individual’s connection to the environment (through wellbeing) (Chaigneau et al. 2022). For example, strong familial and psychological connections to farming in New Zealand bolstered the resilience and adaptive capacity of farmers to the removal of subsidies (economic shocks) and frequent droughts (environmental shocks) (Pomeroy 2015). For sustainable seafood production, research suggests that ethically, equity needs to go beyond intergenerational equity to cover equal access to food, marine ecosystem goods and services (e.g. fish stocks), coastal and marine areas, culturally important areas, species and communities, public services and financial capital from fisheries (Alexander et al. 2021; Bennett et al. 2022). This includes equal share of the economic benefits and impacts of environmental change. Access and involvement in decision-making is needed, with transparency, consultation and knowledge sharing. Lastly, the degree of agency, level of economic capacity, types of knowledge systems used and scoping of fair and just treatment, with dignity and respect (including fair and just systems of law) needs to be considered (Alexander et al. 2021; Bennett et al. 2022). Equity in turn, improves livelihoods and wellbeing by sustaining the economic, cultural, spiritual and social connections between humans and the marine environment (Betley et al. 2021).

Seafood is essential to some Indigenous cultural practices and this may not be accounted for in other perspectives or knowledge systems (Kittinger et al. 2015). Indigenous perspectives view humanity as integrated within the natural world and emphasise the relationship between culture and knowledge, where accumulated knowledge is considered cultural capital and transferred through cultural vectors such as language (Throsby and Petetskaya 2016). Indigenous frameworks focus more on the steady state of the system and emphasise maintenance rather than development and economic growth (Throsby and Petetskaya 2016). These frameworks are also location and society specific, built on notions of shared responsibility (rather than private ownership, Throsby and Petetskaya 2016) and the sacredness of natural resources, which may not be considered in western frameworks (Kealiikanakaoleohaililani and Giardina 2016). First Nations sustainably lived off the land for millennia (Braun 2022) and there is a wealth of knowledge that can be learned and shared through collaborative efforts to build resilience and sustainability (Hale et al. 2022). However, these communities have often suffered major disruptions to their knowledge systems and can be disadvantaged in terms of access to the resources (e.g., support, infrastructure, networks) that enable resilience in western communities. Therefore, adequate support and capacity building (through equity) are also needed to complement Indigenous resilience and sustainability solutions (van Putten et al. 2013).

### Disruptions to seafood supply chain networks

There are many driving factors in the social, environmental and economic dimensions of a SSCN on both land and sea (Fig. 1) (Amos et al. 2022). Recent disruptions (e.g. COVID-19) have emphasised just how detrimental shocks can be to SSCNs and how planning for, and adapting to disruption (i.e., building resilience in conjunction with sustainability goals) can reduce the social, economic and ecological consequences of these shocks (White et al. 2022). Disruptions to SSCNs vary in frequency and intensity; they can occur as a single event or multiple occurrences of the same disruption (e.g., floods, marine heatwaves), a long-term change that culminates in a disruption (e.g., stock collapse) or a co-occurring set of changes to (and within) the SSCN. Categorising disruptions and recording responses can unearth themes to help with response planning. In our search, we found three disruption types that we placed into categories adapted from the ecological perturbation literature.

**Fig. 3:**
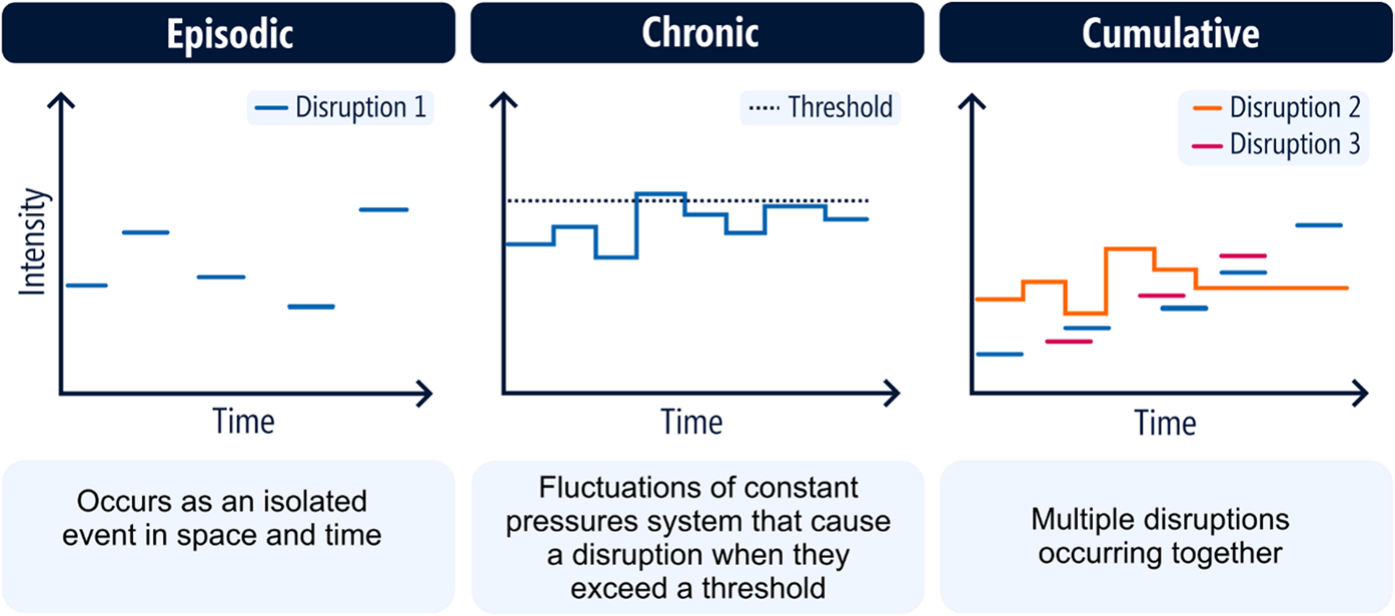
Broad categorisation of disruption types experienced in seafood supply chain networks. Episodic disruptions are distinct from chronic disruptions as they are absent between occurrences

Seafood supply chain disruption categories were adapted from press and pulse perturbations described in ecology (e.g. Harris et al. 2018). Ecological pulse perturbations describe a single disruptive event such as large rainfall events or intense heatwaves (Harris et al. 2018). In the same vein as a pulse perturbation, disruptions to seafood supply can occur as a single event in time, like a flood or an earthquake. We refer to these disruptions as episodic (Fig. 3). Press perturbations are referred to as the long-term changes of a driver like ongoing climate change (Harris et al. 2018)*.* Comparably, SSCNs are connected to regulatory variables, both nationally and internationally, exerting constant pressures on the system that fluctuate over time. When a change in the variable exceeds a threshold, a disruption occurs, which we categorise as chronic (Fig. 3). Episodic and chronic disruptions can and do occur together in time and we refer to these as cumulative (Fig. 3).

### Episodic disruptions

Episodic disruptions describe disruptions that occur as individual events isolated in space and time (Fig. 3). Environmental shocks such as marine heatwaves and floods are examples of episodic disruptions that impact marine ecosystems and can affect every step of the seafood supply chain (Davis et al. 2021). Floods for instance, can introduce contaminants into marine environments (Johnson 2022) and disrupt transport networks (Smith et al. 2016). The impacts of episodic disruptions are most studied at the production end of supply chains, and the most-studied environmental shocks are temperature extremes and climate cycles such as the El Niño Southern Oscillation (Davis et al. 2021). We draw upon previous reviews, particularly reviews of climate related responses detailed in Davis et al. (2021) and Smith et al. (2021) to identify resilience-building attributes and sustainability implications of episodic disruptions (Table 2). We include a case study example (Table 3) to expand on the findings.

**Table 2:** Examples of reported or available responses of seafood supply chain networks to chronic disruptions. N indicates negative outcomes for sustainability and P indicates positive outcomes

Study	Impacts	Response	Resilience attributes	Limitations experienced	Sustainability implications
Fisheries management–industrialised fishing in New England (Tolley et al. 2015)	Led to focus on high value species Stock collapses of favoured species (e.g., cod) Displacement of local coastal communities and fishers Reduced accountability and transparency to the public	Community-supported fisheries established Local supply chain re-established	Collaborative action by local communities, advocates and small to medium scale fishermen	Fishery management policies created economic hardship for small to medium scale fishermen Management did not utilise local fisher knowledge of their fishing grounds to sustain local fisheries Government policies privatising fishing rights	Improved local ecological, economic and social sustainability^P^
Geopolitics–export-oriented salmon producers in Norway (Chen and Garcia 2015; Graziano et al. 2018)	China imposed stringent non-tariff border measures on Norwegian salmon after the Norwegian Nobel Committee awarded the Peace Prize to a Chinese human rights activist	Norway redirected export to existing and emerging markets (including smuggling)	Ability to find new channels for export Norway increased exports to China through these channels	Smuggling introduced competition with other salmon exporters (market distortion) Degradation of salmon quality through delays, improper documentation and reduced Chinese consumer confidence in the product Increased costs for salmon importers in China	Economic sustainability of the industry^P^ Potential rotting of fresh salmon because of delays (wastage)^N^ Impacts on consumer welfare from smuggled salmon^N^
Shifts in the distribution of north-east Atlantic mackerel stocks (Graziano et al. 2018)	International disputes between the European Union, Norway, Faroes and Iceland that led to overfishing and loss of the Marine Steward Council (MSC) certification	International collaboration to get recertified	European fisheries were open to collaboration to get recertified	Lack of cooperation by Iceland	Fishing over quota of Atlantic mackerel stocks^N^ Reduced sustainability efforts through loss of MSC certification^N^

**Table 3:** Impacts of environmental extremes and disease outbreaks on the Pacific oyster supply chain

Introduction The Pacific oyster (*Crassostrea gigas)* was introduced in the 1940s to Tasmania, Australia by CSIRO for aquaculture purposes (Stephens and Myers 2020). It is now commercially grown in New South Wales, South Australia and Tasmania (Schrobback et al. 2020), supplying to the domestic hospitality sector (Ogier et al. 2021) and supporting over 4000 jobs (Stephens and Myers 2020) Oyster production is sensitive to environmental change and water quality. Dips in oyster production have been directly linked to pollutants and nutrient flows into estuaries. For example, flooding in Nambucca River (Australia) decimated oyster stocks and triggered a sewage spill, causing mandatory 21-day closures for oyster growers (Johnson 2022). Ocean acidification and increased temperature can slow growth rates, reduce fertility and increase spat mortality. Drought can lead to high salinity, increasing the occurrence of predatory flatworms that impact oysters (Stephens and Myers 2020) Of the environmentally driven disruptions to production, Pacific oysters are the most vulnerable to Pacific Oyster Mortality Syndrome (POMS), a disease caused by the OsHV-1 virus. POMS is seasonal, occurring during the summer months (NSW Department of Primary Industries 2020) however, once triggered, it can spread quickly and cause rapid mortality (Department of Primary Industries and Regions 2019) Disruption POMS was first seen in New South Wales in 2010 after which movement of oysters and oyster products was restricted to avoid further spread of the disease. In 2015–2016, the longest and most acute marine heatwave ever recorded occurred in the Tasman Sea. This led to the first outbreak of POMS in Tasmania (Oliver et al. 2017). POMS spread to five oyster growing areas, causing up to 95% oyster mortality (IMAS 2019). Close to one third of Tasmanian oyster cultivators were affected by the virus (Ugalde et al. 2018). South Australia banned live Pacific Oysters, oyster spat and farming equipment from Tasmania. This resulted in a shortage of spat in South Australia (and New South Wales) who acquire 90% of their spat from Tasmania (Department of Primary Industries and Regions 2019) Supply chain response To remediate this issue, the South Australian government provided support to two small existing hatcheries and to develop two new hatcheries in South Australia to increase production, although issues of reliability and quality persist (Schrobback et al. 2020). Two of Tasmania’s largest spat producers also opened hatcheries in South Australia (Wan 2018; Nogrady 2019) and the Australian Seafood Industry established a breeding centre in South Australia to supplement spat supply from Tasmania (Schrobback et al. 2021) Preliminary insights from research on POMS enabled Tasmanian businesses to act quickly to establish bio-secure facilities, such as the installation of water filtering equipment, killing viruses with UV light and adding cultured phytoplankton to the water (Catizone 2016). This enabled some Tasmania hatcheries to continue their operations and supply in Tasmania (Nogrady 2019). Factsheets were available to inform growers on how to sanitise equipment and continue supply (Catizone 2016; Oyster Health Sydney 2016) Research also identified handling and management practices that reduced mortality such as selling before the warm summer weather (Nogrady 2019). Size is also an important factor, since smaller oysters are more at risk than larger oysters, and farmers can vary farm management practices in order to regulate growth rate (Ugalde et al. 2018). This means hatcheries are more at risk, but a team of scientists developed new methods to protect spats which have already proven successful (FRDC 2016). Additionally, selective breeding and testing is leading to the development of POMS resistant pacific oysters (Stephens and Myers 2020). Monitoring is very important to anticipate and adapt to any potential change in environmental conditions that might disrupt or trigger an outbreak (e.g., changes in water temperature, nutrient levels or chlorophyll concentration). Technology is making monitoring of water quality in oyster-producing estuaries more accessible and affordable (Stephens and Myers 2020) Lessons learned for resilience and sustainability **Resilience** • Importance of spatial diversity in hatcheries for maintaining supply when hatcheries in one location are disrupted • Research and experimentation to investigate causes of POMS, test protection measures that reduce mortality (handling and management) and develop POMS resistant stocks (breeding) • Support from state government and industry assisted the development of new hatcheries and existing hatcheries to maintain production • Sharing of POMS research internationally and within Australia to enable adaptive capacity across hatcheries and the industry overall Despite the POMS outbreak, a survey concluded that 79% of Tasmanian oyster businesses still considered their oyster farm operation as strongly viable (Ugalde et al. 2018). It was believed this confidence was due to the support offered by the government and industry representatives, as well as lessons learned from other regions which had been previously affected by the disease such as France and Spain (Ugalde et al. 2018). Some farmers have suggested that the industry has become more resilient as a result (Nogrady 2019) **Sustainability** The Pacific oyster industry supports rural coastal communities and economies (Schrobback et al. 2020). Increased collaboration between farmers, industry representative and governments will support the continuation of these businesses under disruption. The sensitivity of oyster production to pollution, contaminants, water temperature and algal blooms can act as an indicator of ecosystem health (Hick 2020) and be used to improve habitat availability for wild fisheries and buffer against storms (Stephens and Myers 2020)	

General themes emerge within the reported and proposed food supply chain responses to environmental shocks (Davis et al. 2021). In food (and seafood) production, diversifying harvesting methods or species, accessing subsidies, shifting to resistant breeds, relocating businesses or harvesting regions and investment in research and development have been used or proposed to combat environmental shocks (Lim-Camacho et al. 2014; Davis et al. 2021). Strategic reserves and primary processing methods such as solar drying can enhance processing capacity and increase the shelf-life of seafood, averting food insecurity. Similarly, trade agreements to source product from regions unimpacted by disruption can add functional redundancy to a supply chain. Retail and markets can maintain business by promoting seafood products and encouraging diet shifts. Subsidies can also encourage consumers to make more nutritious choices. Across supply chain stages, investment in infrastructure for monitoring (warning systems), equipment (e.g., boats), research, transport (e.g., roads), storage (especially cold storage) and markets is highlighted as important for being resilient (Davis et al. 2021). Climate shocks may increase risks for foodborne illnesses, which can be lessened through strengthening food safety regulations and/or research into disease and climate-resistant species (FAO Climate Change 2020; Davis et al. 2021).

Seafood production is particularly vulnerable to marine heatwaves (short-term warming events in the ocean) (Mehrabi et al. 2022). Around the world, marine heatwaves have resulted in stock declines, harmful algal blooms, mass mortalities, economic losses and fisheries closures (due to low catch and recruitment). Increasing temperatures and carbon emissions are increasing the frequency and intensity of marine heatwaves (Smith et al. 2021) and having plans in place to cope with these disruptions can significantly increase resilience. In the Gulf of Maine, learning from the way a marine heatwave transferred through the supply chain and implementing adaptations led to economic gains during the next marine heatwave (Pershing et al. 2018). The 2015–2016 marine heatwave in Tasmania triggered an outbreak of the Pacific Oyster Mortality Syndrome, causing mass mortalities across farms and halting the supply of spat to other states in Australia. However, research on previous outbreaks of POMS both nationally and internationally and testing mitigation approaches led to a quick recovery despite the unavoidable losses (Table 3).

### Chronic disruptions

Chronic disruptions result from changes in the longer-term influences on SSCNs that extend beyond the threshold and trigger a disruption. This could be fluctuations in regulating variables (i.e., the slow variables in Table 1) such as fisheries management, geopolitics, market demands, consumer preferences, labour or climate cycles and changes to harvested stocks (Gephart et al. 2017; Graziano et al. 2018). Overfishing (or mismanagement) of marine resources and geopolitical crises (e.g., breakup of a country) have been identified as frequent causes of shocks to seafood production (Gephart et al. 2017; Cottrell et al. 2019). Table 2 identifies resilience-building attributes and sustainability implications from three examples of chronic disruptions reported in the literature. Responses suggest that collaborative action both locally and internationally with an emphasis on ecological sustainability is required for resilience to chronic disruptions. Although other studies also indicate that altering volumes of imports and exports improve short-term resilience (Gephart et al. 2017).

A shift to industrialised fishing in New England led to a focus on high value species. This improved the economic viability of the New England cod fishery but impacted ecological and social sustainability through overfishing and displacement of local communities (Table 2). Collaborative action by local communities re-established the local supply chain and reduced fishing pressure on high-value species. Overfishing has been tied to geopolitical tensions. For example, Canada prohibited French boats from fishing in shared cod fishing grounds upon claims of France exceeding their quota. This led to a drop in fish catch, potentially disrupting associated supply chains and livelihoods for French fishers. Subsequent overexploitation in the same region resulted in the near commercial extinction of cod stocks (Gephart et al. 2017). The fishery was closed to rebuild stocks and imports were increased to compensate, impacting livelihoods and related supply chains in both countries (Gephart et al. 2017). Salmon exports in Norway were disrupted by stringent border measures upon arrival in China, prompting Norway to find alternative routes to China. However, this led to a drop in quality, consumer confidence in the product and wastage of salmon (Table 2).

Shifts in the distribution of north-east Atlantic mackerel stocks prompted international disputes between the European Union, Norway, Faroes and Iceland that led to overfishing and loss of the Marine Steward Council certification (Table 2). International collaboration was required to get re-certified. As climate change (and other chronic disruptions) continues to disrupt SSCNs, planning and collaboration is needed to reduce the chances of conflicts that negatively impact marine resources and dependent communities. For example, climate-driven redistribution of tuna stocks may disrupt incomes for Pacific Island countries and territories through reduced access fees. This also has implications for the sustainable management of the purse-seine tuna fishery as the fishery operates under regulations set by cooperative management between member island countries and states (Bell et al. 2021). Collaborative efforts to implement alternative policies will be necessary to sustain tuna-dependent economies in the Pacific Islands and fisheries management in the high seas (Bell et al. 2021).

### Cumulative disruptions

Cumulative disruptions describe disruptions that coincide with other disruptions (Fig. 3) (Mehrabi et al. 2022). The COVID-19 pandemic is a prime example. To reduce rates of infection, governments around the world introduced distancing measures (e.g., 1.5 m guideline), curfews, lockdowns, border closures and protective gear such as masks. Most SSCNs continued to supply seafood to consumers despite the limitations imposed on fishing and aquaculture operations. As such, COVID-19 responses provide invaluable information on how SSCNs can adapt to cumulative disruptions (Stoll et al. 2021; Bassett et al. 2021). Table 4 summarises how COVID-19 has impacted SSCNs around the world, how supply chain actors have responded and identifies the resilience-building attributes applied, the limitations experienced, and the positive and negative implications of those responses on sustainability.

**Table 4:** Reported responses of seafood supply chain networks to COVID-19 disruptions. Note that this is a select list of studies of regions around the world that documented COVID-19 responses. N indicates negative outcomes for sustainability and P indicates positive outcomes

Study	COVID-19 disruptions	Response	Resilience attributes	Limitations experienced	Sustainability implications
Seven small-scale fisheries from Indonesia, India, Peru and USA (Bassett et al. 2021)	Loss of export markets Reduced local demand due to financial insecurity	In all fisheries, shifted supply to local and regional distribution channels by leveraging social networks, building local networks, and using technology to market (e.g., social media) Fishers consumed unsold fish Reduced business costs (frequency of fishing trips and fish catch) Promote in-home consumption of seafood	Utilising existing local or regional distribution pathways to get product to local consumer or business (e.g., shift from restaurant to retail)	Lack of storage No bureaucratic support (e.g., fisheries not considered an essential business) For exported products, there was no local market for product or connection with consumers Money to cover reduced income and business costs Lowered prices for seafood Barriers on minimum price due to trip costs	Financial instability for fishers and consumers through loss of income^N^ Shift in consumer demand for less expensive proteins (e.g., chicken)^N^ Fish wastage^N^ Reduced fish catch (potential positive outcome)
USA and Canadian alternative seafood networks–direct to consumer supply (Stoll et al. 2021)	Increased demand for local and directly sourced food	Increased supply to meet demands	Strong harvester-consumer relationships Strong relationships across the supply chain (e.g., processors, seafood community organisations) Diverse fishing seasons, subscription services, delivery options and ordering methods to suit consumer needs Control over pricing and access to financial capital Psychological resilience Online marketing platforms were already in place	Processing or transport infrastructure Price uncertainty from processors and buyers	Repurposing existing channels to distribute seafood direct to consumer under disruption^P^ Adapting prices to increase accessibility for consumers also undergoing financial stress (i.e., increasing equity)^P^ Valuing sustainability and human wellbeing alongside profits (Witter and Stoll 2017)^P^
Australian seafood industry (Ogier et al. 2021)	Reduced dine-in and increased retail market Loss of export market Increase in import-competing and retail products Increase in price due to high domestic demand	Redirect product from dine-in to retail Shift from export to domestic markets and bulk frozen food to retail Establish or expand online sales Production inputs sourced locally Export products shifted to value-add or packaging Reduced domestic road and air freight limiting regional and rural producers (e.g., farmed abalone, mud crab, barramundi)	Government (federal, state and territories) labelled fisheries and aquaculture as essential services and provided financial support Knowledge sharing between government agencies (e.g., Australian Fisheries Management Forum) Versatile and larger firms were able to absorb more disruptions	Smaller businesses may have limited adaptive capacity	Ongoing financial support interventions may not be economically and/or socially sustainable in the longer term
Small-scale fisheries and coastal communities (Bennett et al. 2020)	Loss of export market, tourism and access to cold storage Port closures No shipping or air freight Reduced demand from local restaurants and hotels Factories operating at reduced capacity or closed Migrant fishers stranded on boats Women impacted by post-harvest disruptions and at higher risk of exposure to COVID-19 Financial insecurity and shortage of basic needs Exclusion from government relief	Fishers and communities sourcing and sharing food (mostly for free) Adapting distribution models and using online platforms to deliver seafood to households	Strong community action amongst small-scale fishing communities Government listening to the community	Fisheries not considered an essential business Social distancing limited fishing opportunities and selling at markets Limited cold storage	Fish waste due to limited freezer storage^N^ Maintaining food security for rural communities without access to markets^P^ COVID-19 compounded impacts for disadvantaged communities^N^ Increase in illegal fishing as reduced capacity to patrol this activity^N^ Fishers working together to assert rights to continue to fish and provide food under safe working conditions^P^ Opportunities to improve working conditions for migrant workers (Marschke et al. 2021) and support for women (International Organisation for Women in the Seafood Industry 2020)^P^ Reduced fishing pressure on ecosystems^P^
Northeast USA commercial fishers (Smith et al. 2020)	Loss of export and domestic restaurant markets Decrease in seafood price Reduced income Loss of processing capacity	> 50% of fishers continued fishing despite loss of income Harvested other species (e.g., more marketable species) Sold and delivered directly to consumers	Ability to switch species or change fishing location or trip length Revised fisheries regulations allowed for more direct sales to consumers Financial support from the government to supplement income loss and reduce exposure to COVID-19 Initiatives to promote direct sales of seafood	Buyers had limited freezer space Tariffs on seafood export to China Limited crew due to social distancing rules	Increased harvesting pressure by focussing on more marketable species^N^ Long-term livelihood of fishers under disruption^N^ Fisher health and wellbeing–risking exposure to COVID-19 by continuing to fish^N^
Aquatic food value chains in Bangladesh, Egypt, India, Myanmar and Nigeria (Belton et al. 2021)	Experienced temporary dips in ability to buy inputs, sell product, find buyers, access transport, employment of casual workers Temporary difficulty finding workers and employment for women more impacted Reduced demand led to reduction in trader’s sales prices, fish sold and income Increase in price of feed due to increased operation and transport costs Food insecurity Prices of products sourced from global markets remained stable, reducing profit margin for local businesses	Sought supplemental income as labourer, used savings, borrowed money or sold their labour Individuals purchased less food than usual, skipped meals or grew their own High-income earners (e.g., operators of large hatcheries or feed mills) shopped online for groceries and adhered to food safety guides Paused business operations, minimised operating costs, bulk buying inputs, sourcing alternative inputs (often lower quality), selling product at discounted rates, borrowing capital, paying bribes to continue operations Selling product online, offering delivery, diversifying operations, selling direct to customers, coordination across hatcheries to set a minimum price, expanding trade credit to maintain demand	Some relief (e.g., food, money) provided by larger businesses to smaller businesses, workers and neighbours Utilisation of online platforms to source food, market product and find consumers Supportive relationships between businesses Some financial support from government	Lower-income groups, small-scale farmers and fishers had limited options to supplement loss of income. They also did not report carrying out food safety practices (possibly due to limited access to resources) Limited financial assistance to individuals from the government (except for India) Unequal allocation of support funds Lack of information on governmental financial assistance (Myanmar) Confusion around transport exemptions (information sharing, Bangladesh)	Wellbeing of workers facing uncertainty, inability to find work and loan repayments^N^ Nutrition and food insecurity for low-income earners and increased vulnerability to COVID-19^N^ Potential exploitation of smaller businesses, workers and neighbours by large businesses offering relief^N^ Persistent reductions in demand after COVID-19 like disruptions^N^ COVID-19 compounded impacts for already disadvantaged communities (e.g., Bangladesh) especially for women^N^ Securing seafood supply to the Global South provides affordable nutrient rich food, sustains local economies and livelihoods and is an investment opportunity for governments and development agencies through regular consultation with industry and equitable allocation of resources Potential overharvesting of catch under disruption^N^

SSCNs including small-scale fisheries and coastal communities experienced reductions in demand for seafood due to the absence of a local market for a primarily exported product, affordability or reduced restaurant markets (Table 4). Declining demands led to markets in France, Japan, Mexico, Spain and Portland (USA) experiencing between a 19 and 51% price drop of seafood product with variations of up to 79% from the 5-year average and some price drops persisting until the end of 2020 (Amos et al. 2022). COVID-19 restrictions and trade bans also culminated in losses of export markets, reduced labour or facilities to transport, store and process seafood and reduced ability to fish. Fishers and supply chain actors sought alternative ways to market, produce, process and distribute their product. Indigenous fishers like the Torres Strait Islanders have limited alternatives and had to absorb the financial consequences with negative impacts to socio-economic sustainability (Plagányi et al. 2021).

In most cases, harvesters and fishers supplied seafood directly to consumers, shortening the supply chain (Table 4). Supply was shifted to local or regional communities through existing distribution channels and by leveraging or developing strong relationships with consumers. Fishers also switched target species or fishing seasons and used online platforms to market and sell product (Table 4). Export dependent SSCNs needed to first develop a local market to distribute product (Table 4).while SSCNs with existing local markets experienced an increase in demand (Stoll et al. 2021). Government assistance either financially or through policy changes (e.g., labelling fisheries as an essential business) and knowledge sharing between communities and governments aided the adaptive capacity of SSCNs. Moran et al. (2020) suggest that supermarkets in the UK were able to withstand shocks in demand as access to infrastructure, logistics and healthy profit margins enabled retailers to bear higher costs in order to maintain food supply. This was also a key adaption for Australian SSCNs (Table 4).

These responses highlight vulnerabilities for already disadvantaged communities and countries. SSCNs and communities lacking in governmental assistance, information sharing and infrastructure experienced more negative impacts, especially for women and migrant workers (Table 4). This led to maladaptive responses that compromised the health and wellbeing of individuals and communities (e.g., skipping meals, Table 4). Small-scale businesses were also susceptible to exploitation by large-scale businesses. In west Africa, COVID-19 compounded the effects of other disruptions, such as hunger, conflict and climate change (Bennett et al. 2020).

## Attributes of resilient seafood supply chain networks

Shocks to production, processing, storage, distribution and markets were seen across all three disruption types. Consistent responses were also seen across disruption types, with specific responses seen for episodic disruptions (i.e., breeding and research and development). We looked for characteristics within responses that represented the attributes in Table 1. For example, if a SSCN used alternative options for harvesting or transport, then the SSCN was considered to have an element of diversity, which enabled its resilience. Across disruptions and responses, we find that diversity, connectivity, collaboration, learning and polycentric governance are the main attributes that enabled resilience to all types of disruption in seafood supply systems. Table 5 presents these attributes with examples from Tables 2–4 and the case study in Table 3. These can be applied to individual businesses or across SSCNs at local and global scales, though increasing scale will require more emphasis on collaboration and learning.

**Table 5:** Attributes of resilient seafood supply chain networks and examples of use as described in this review (see Table 2, Table 3 and Table 4). Examples are ordered by timescale needed for implementation. “ST” indicates short-term (days to months) and “LT” long-term (years to decades)

Attribute	Examples of attribute use to enhance seafood supply chain resilience (from Tables 2, 3 and Table 4)
Diversity	• Shifting markets and consumers (restaurants to retail or international to local consumers, Ogier et al. 2021)^ST^ • Diverse fishing seasons, shift fishing grounds and/or target species (e.g., Smith et al. 2020)^ST^ • Building financial capital to increase flexibility under disruption (e.g., flexible prices, Stoll et al. 2021)^LT^
Connectivity	Connectivity of the SSCN: • Distribution pathways (e.g., to divert product from restaurant to retail, Bassett et al. 2021)^ST^ • Proximity or awareness of supply chain to consumers (e.g., Stoll et al. 2021) ^ST, LT^ • Strong producer–consumer relationship (e.g., through communication or information sharing, Stoll et al. 2021)^LT^ • Proximity of producers to next stage of supply chain (Table 3)^LT^ • Building trust-based relationships among supply chain stakeholders (including governing bodies) (e.g., Bennett et al. 2020)^LT^
Collaboration	• Collective action (Tolley et al. 2015)^ST^ • Trade agreements (Graziano et al. 2018)^LT^ • Building trust-based relationships among supply chain stakeholders, including governing bodies (Table 3)^LT^
Learning	• Information sharing within and across supply chain and with government (through trust-based relationships, Table 3)^ST^ • Learning from past disruptions to develop a response strategy (Pershing et al. 2018)^ST, LT^ • Adopting new ways to market or find customers (e.g., online platforms, Belton et al. 2021)^ST^ • Research and development (breeding climate or disease resistant species, Table 3)^LT^
Polycentric governance	Support from governing bodies, for example: • Changes to fisheries management regulations (e.g., Smith et al. 2020)^ST^ • Labelling fisheries as essential businesses (e.g., Ogier et al. 2021)^ST, LT^ • Financial support from governing bodies (Table 3)

Diversity provides options for supply chain actors to respond to a disruption, which largely enhances the flexibility of communities, businesses or whole supply chains. For producers, this may be diversifying harvested species (Table 4) and for supply this may be having more than one transport route, or diversifying clientele (Table 5). Connectivity within and across stages of the supply chain enforced strong trust-based relationships that enabled resilience under disruption. Similarly, proximity to consumers enabled continued seafood supply by developing or utilising existing connections between producers and consumers (Table 5). This shortened the supply chain and improved resilience. Collaboration enhances these relationships, and by extension resilience, when supply chain actors work together to adapt to disruption (Manlosa et al. 2021). Collaborative action bridges compartmentalised knowledge (Cockburn et al. 2020), increases information sharing, trust and strengthens learning (Table 5). Lastly, participation from all levels of government (polycentric governance) was crucial to the resilience of many SSCNs under disruption (Table 5). We found that government intervention in the form of changes to fisheries management and policy helped supply chain actors adapt. This suggests that resilience is enhanced when boundary-setting organisations are working together with supply chain operators. Subsidies also supported adaptive responses however, continued reliance on subsidies could encourage non-resilience (Ogier et al. 2021). Strategic subsidies could improve both resilience through financing research and breeding programs, promoting sustainable seafood products and practices, and removing subsidies that support non-resilient practice (Ward et al. 2022).

While these responses were largely ad-hoc, they are useful for understanding SSCN adaptation options and the adverse consequences of some responses for sustainability. Consequences included the unequal treatment of supply chain actors, increased vulnerability to exploitation of labour, and overfishing of stocks. There is a risk that if responses remain ad-hoc, continued negative impacts could lead to maladaptive responses such as piracy, human trafficking and hunting in nature preserves (Gephart et al. 2017). Additionally, responses will require varying levels of time and effort to implement (Table 5). For instance, acquiring new customers or adjusting harvesting activities are short-term responses compared to developing flexible trade agreements to facilitate resilience. Slow variables (e.g. climate related shifts to species distribution) may take time before they impact the supply chain, or may be slow to travel up the levels of governance as disruption is happening, requiring awareness and planning to respond (Novak et al. 2021; Davis et al. 2021; Amos et al. 2022). Investing in planned responses has significantly improved supply chain resilience and benefitted supply chain actors (see episodic disruptions and Table 3). However, additional work is needed to discourage responses that compromise ecological, sociological and economic sustainability (Love et al. 2021; Ruiz-Salmón et al. 2021).

## Implications for socio-ecological sustainability

**Fig. 4:**
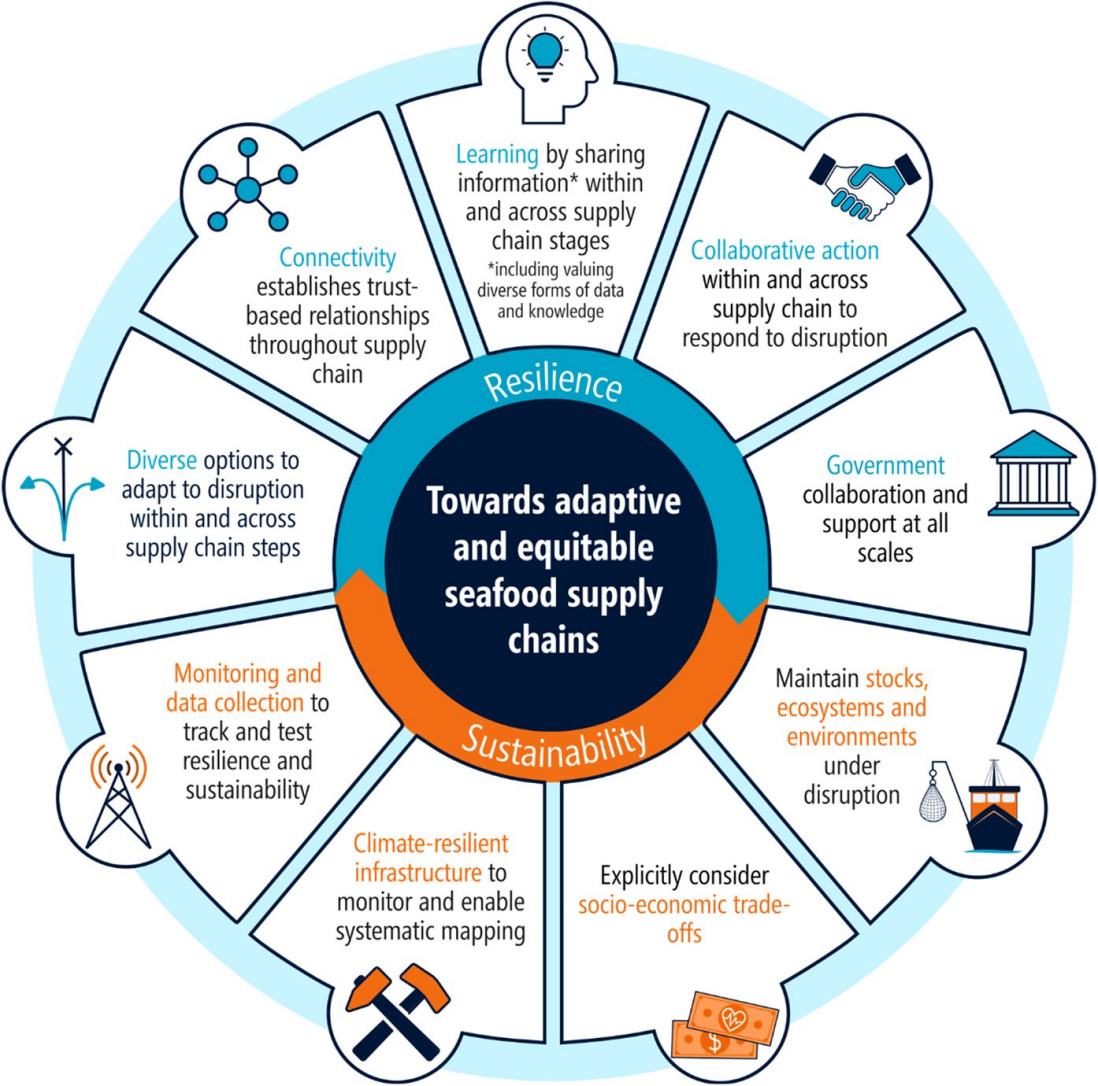
Needs for adaptive and equitable seafood supply where resilience enables sustainability and reduces negative feedbacks

From our findings, we outline focus areas for improving socio-ecological sustainability and refer to the literature for potential solutions. The five key attributes we have identified for building resilience in SSCNs (diversity, connectivity, collaboration, learning and polycentric governance) are also important for improving sustainability (Fig. 4). Recent visions for a sustainable seafood system place collaboration and learning, through trusted relationships, as vital needs for key actions with diversity and connectivity as operational elements (e.g., Melbourne-Thomas et al. 2021; Trebilco et al. 2021; FAO 2022; Farmery et al. 2022; Mehrabi et al. 2022). Additionally, as governing bodies and guidelines (e.g., food safety, private food standards, fisheries and aquaculture management, biosecurity or trade guidelines) set the boundaries that socio-ecological systems operate within, implementations to support sustainability need to occur in collaboration with governance (Love et al. 2021; Nash et al. 2022b; Mehrabi et al. 2022). Our study supports this as collaboration and learning were found to build resilience while human conflict was found to disrupt seafood supply and/or led to overfishing. If not addressed early, fisheries eventually close to rebuild stocks and seafood is imported to compensate (Table 4), potentially increasing pressures on external stocks and ecosystems (Klein et al. 2022). Ecosystems and fished stocks are not often connected to the rest of the supply chain in food system analyses (Simmance et al. 2022). This results in a gap in the understanding of the socio-economic and cultural sustainability implications of anthropogenic impacts to marine resources that feedback into the supply chain (Ahmed et al. 2019; Farmery et al. 2022; Mason et al. 2022). Table 6 summarises the focus areas for improving SSCN sustainability, which can be achieved by utilising resilience building attributes (Fig. 4).

Global demands for food security, health, and wellbeing can be met in part by sustainably managed marine ecosystems, provided current concerns are addressed (Merino et al. 2012) (marine environmental sustainability, Table 6). The suite of concerns impacting marine environmental sustainability include warming (Trebilco et al. 2021), marine biodiversity (Ward et al. 2022), pollution (FAO Climate Change 2020), animal welfare (Lam 2019), foodborne disease outbreaks (FAO Climate Change 2020), species redistribution (Melbourne-Thomas et al. 2021), seafood packaging (Almeida et al. 2022) and fisheries and aquaculture impacts (Ahmed et al. 2019). Existing coastal and ocean management systems are fragmented (e.g., fisheries, aquaculture, recreation, transport) (Stephenson et al. 2019). With collaboration and learning (including sharing of innovations, Table 3), holistic fisheries and aquaculture management can be planned for and implemented prior to disruption (Farmery et al. 2022; Mason et al. 2022). Improved management of marine environments and resources could utilise underfished resources, reduce discards, minimise waste and other environmental impacts of fishing and aquaculture (e.g., loss of fishing gear, carbon footprints, habitat loss) (Ahmed et al. 2019; FAO 2022). Stephenson et al. (2019) propose linking and adapting existing (siloed) management systems into an overarching program that involves a shared vision, common operational objectives, collaborative decision-making through appropriate legal and institutional frameworks, flexibility to change, explicit consideration of trade-offs and cumulative impacts, and effective and iterative processes for stakeholder participation and evaluation. Similarly, Froehlich et al. (2021) suggest an iterative holistic approach to fisheries management, that is supported by data, integrates wild fisheries and aquaculture and balances socio-ecological trade-offs (e.g., Finkbeiner et al. 2018).

**Table 6:** Focus areas for seafood supply chain network sustainability that can be achieved with resilience building attributes

Focus area	Improvements needed to address sustainability outcomes from seafood supply chain responses to disruption	Emerging
Marine environmental sustainability	Holistic fisheries and aquaculture management to: Reduce chances of overfishing under disruption Better utilise marine resources Minimise waste Adapt to climate change (including shifting distributions) Maintain marine biodiversity	Minimising environmental impacts of fishing and aquaculture production practices Decarbonisation of production / distribution Increasing animal welfare Sustainable packaging
Socio-economic sustainability	Explicitly consider trade-offs between social, economic and cultural sustainability in planning Prioritise health, equity and wellbeing under disruption: o Consideration of cultural priorities and indigenous aspirations o Fair treatment of workers (e.g., no slavery) o Gender and age equality o Access to food security and nutrition	Stakeholder and consumer awareness of resilience and sustainability (through collaboration and learning) to increase demand for sustainability Opportunities for a circular economy Stock redistribution increasing distance between centre of stock and location of landing/processing facilities Increase consumption of locally produced products (provenance)
Infrastructure sustainability	Resilient infrastructure to assist seafood supply chain resilience and sustainability through: Alternate transport routes or modes Processing and cold storage facilities (e.g., Miles 2023) Research facilities Monitoring and data collection	Infrastructure development and planning not climate-ready Coastal processing facilities are threatened by climate change Stock redistribution increasing distance between centre of stock and location of landing/processing facilities Decarbonisation of transport routes
Monitoring and data collection	Through systematic mapping: Data collection to support modelling and holistic supply chain management Monitoring and data collection to support legislation (e.g., food safety) Networking and making sure all the components fit together Further build resilience through monitoring of slow variables and feedbacks and data collection of multiple drivers	Modelling and scenario testing using collected data Indicators of resilience and sustainability (e.g., Marine Stewardship Council certification) Traceability Provenance–knowledge of where the seafood came from Monitoring compliance

Health and wellbeing suffer when SSCNs are under-prepared for disruption. Table 4 describes instances where the health and wellbeing of supply chain actors was reduced to maintain financial stability. Disruptions also compromised livelihoods and businesses resulting in labour shortages, larger power imbalances and unequal treatment, changes in seafood prices and consumption and disrupted transport routes (Table 4). These impacts were particularly clear in small-scale fisheries, which comprise half of the world’s seafood production and sustains livelihoods for over 90% of global fishers in coastal communities (Knight et al. 2020). Co-producing approaches with stakeholders is essential for establishing context, increasing equal access to information, deploying holistic approaches that will be used and addressing power imbalances in trade through reprioritisation (Mason et al. 2022; Nash et al. 2022a) (socio-economic sustainability, Table 6). Boosting equity through policy will be important for reducing inequities and increasing resilience and sustainability (Hicks et al. 2022). Small-scale and indigenous fisheries can be empowered through government participation and policies and equal access to knowledge (Lowitt et al. 2020). Attachment to place can motivate communities to adapt to disruption but it can also limit their adaptive capacity (Plagányi et al. 2021; Mason et al. 2022). Phelan et al. (2022) suggest options for creating synergies between western and traditional systems for sustainable seafood production. Jurisdictional approaches using place-based incentives that align with government, market and producer incentives can drive resilience and sustainability in these regions (Kittinger et al. 2021).

SSCNs, especially those connected to global trade networks, can be highly influenced by market demands (e.g., Crona et al. 2016). Under disruption, loss of exports negatively impacted SSCNs actors as they searched for income (Table 4). Planning can reduce some of these negative impacts and improve sustainability by collaborating to identify socio-ecological trade-offs (socio-economic sustainability, Table 6). For example, Avadí and Fréon (2015) compared environmental impacts, job opportunities, nutritional profiles and profits provided through different ways of processing anchovies to identify trade-offs. The Australian edible oyster industry is an example where seafood production can improve environmental sustainability, support livelihoods and coastal communities (Table 3). Regional SSCNs (e.g., alternative seafood networks and community-supported fisheries) were more resilient as they had more financial capital and agency over how they can supply and price seafood (Table 4). Localising seafood supply strengthens regional economies, health benefits, increases provenance (Watson et al. 2016), reduces carbon footprints, and decreases reliance on global trade in places such as the Pacific (Farrell et al. 2020; Ruiz-Salmón et al. 2021). Export SSCNs can adopt market-based approaches (e.g., certifications, buyer commitments and fishery improvement projects) that integrate more social responsibility to mitigate violations of human rights, provided they are not voluntary, regularly monitored for compliance and have mechanisms in place to address non-compliance (Lout 2023). There are additional opportunities to improve sustainability by using resilience attributes to implement a circular economy (Fletcher et al. 2021) or vertically integrate (Davis et al. 2021). SSCNs are also influenced by consumer demands therefore, educating the consumer on SSCN sustainability, the seasonality of seafood and being transparent in SSCN operations can increase provenance (Watson et al. 2016) and empower consumers to make choices that lead to more adaptive and equitable SSCNs (van Putten et al. 2019; FAO 2022).

Infrastructure is emphasised in most SSCN responses as necessary for continued resilience and improved sustainability (Infrastructure sustainability, Table 6). This is also highlighted in other research (Trebilco et al. 2021; Farmery et al. 2022; Mason et al. 2022; Mehrabi et al. 2022). However, is it a costly investment. Prioritising investments could be one way to support resilience and sustainability. For example, infrastructure for cold storage may be of a priority for SSCNs that deal with live or frozen product compared to others. Infrastructure will need to be climate-resilient, especially in coastal regions (Nash et al. 2022b). Existing infrastructure is already undergoing damage from extreme weather events, coastal urbanisation and sea level rise (Trebilco et al. 2021). Shifting species distributions or relocation to climate-resilient areas may increase the distance between stages of the supply chain. For example, harvesting activities occurring further away from processing facilities. This may also increase their vulnerability to delays. Well-planned infrastructure could service more than one domain in need of similar facilities (e.g., transport infrastructure servicing food supply and health sectors) and be set up to collect data. Data is a high priority across all SSCNs for systematic mapping (Farmery et al. 2022; Simmance et al. 2022), holistic management (Froehlich et al. 2021; Mason et al. 2022; Mehrabi et al. 2022), forecasting and responding to disruptive events but will require funding, government support and infrastructure in key nations (Mehrabi et al. 2022). Improved infrastructure and data collection will in turn enable developments in research and/or technology to implement traceability, certifications of equity and sustainability, strong food safety regulations and build trust between SSCN actors and consumers (McClenachan et al. 2016; Roheim et al. 2018; Davis et al. 2021).

Understanding how SSCNs operate as a socio-ecological system helps to identify vulnerabilities and enables collaboration and shared learning during or after disruption (Armenia et al. 2022; Saisridhar et al. 2023). This is critical for capturing feedbacks, changes in slow variables and examining the effects of multiple drivers across each sustainability dimension (Simmance et al. 2022). Network models show promise for developing system-level tools and insights to measure and test SSCN resilience for decision-making (Mehrabi et al. 2022) as they can encompass socio-ecological interactions at multiple scales based on conceptual understandings of the system (Windsor et al. 2022). Approaches to identify these interactions are already part of socio-ecological resilience toolkits and assessment frameworks (e.g. Bergamini et al. 2014) therefore, network modelling can be a useful next step and may be easier to communicate or use for decision-making. The resilience-building attributes described in Table 5 can be modelled through network structures and developed into quantitative indicators. Connectivity for example, has been used to strengthen shipping container networks (Pan et al. 2022) and calculate resilience in seafood supply chains (Plagányi et al. 2014, 2021). There is potential for methods to be applied universally across SCCNs (Lim-Camacho et al. 2017) and metrics can be recorded before, during and after a disruptive event (e.g., Carlson et al. 2021). Further, advances in network modelling such as multi-layered networks and progress towards fully articulated socio-ecological network models demonstrate utility for capturing interactions and feedbacks across scales (Windsor et al. 2022 and references therein).

## Conclusion

Our study contributes to the need for systematic mapping and understanding of supply chain attributes that confer resilience and improve sustainability (Fig. 4). Our findings underscore the need for objective methods for analysing supply chain resilience and points to the need for additional broader tools to better characterise supply chain performance. Seafood supply chains are more vulnerable than other supply chains as they handle live or frozen products with finite shelf lives, require special handling of products and are largely driven by seasonal supply and demand. With cumulative disruptions on the rise, ad-hoc responses can no longer be the default. Food security is a growing issue and while there are caps to global production (Merino et al. 2012), past responses to disruptions (including the COVID-19 pandemic) provide a momentous opportunity to learn and build resilience into holistic seafood supply chain management and planning to meet future demands (Fig. 4).

## Data Availability

All data generated or analysed during this study are included in this manuscript.
